# Datasets for next-generation sequencing of DNA and RNA from urine and plasma of patients with prostate cancer

**DOI:** 10.1016/j.dib.2016.12.016

**Published:** 2016-12-14

**Authors:** A.S. Nikitina, E.I. Sharova, S.A. Danilenko, O.V. Selezneva, T.B. Butusova, A.O. Vasiliev, A.V. Govorov, E.A. Prilepskaya, D.Y. Pushkar, E.S. Kostryukova

**Affiliations:** a Federal Research and Clinical Center of Physical-Chemical Medicine, Moscow, Russia; b Moscow Institute of Physics and Technology, Dolgoprudnyi, Russia; c Department of Urology, Moscow State Medical Stomatological University, Moscow, Russia

**Keywords:** Transcriptome, RNA-seq, Targeted DNA sequencing, Prostate cancer, Benign prostatic hyperplasia

## Abstract

Current prostate cancer (PCa) diagnostic tests suffer from insufficient sensitivity and specificity. Novel biomarkers that can be detected by minimally invasive methods are of a particular value. Here we provide two datasets. The first one is on the whole transcriptome profiling by RNA-seq of urine and plasma obtained from patients with PCa and benign prostatic hyperplasia (BPH). The second one represents targeted sequencing of DNA from urine and plasma of patients with PCa and BPH. Both datasets are available at NCBI Sequence Read Archive under Accession No. SRP093707 and No. SRP093842 respectively.

**Specifications Table**

**Table:** 

Subject area	Biology
More specific subject area	NGS, Transcriptomics
Type of data	Text (FASTQ sequence files)
How data was acquired	High-throughput sequencing using Ion Proton System (Life Technologies)
Data format	Raw
Experimental factors	Urine samples were obtained after prostate massage. Each urine and plasma sample was collected either before or after the operation (radical prostatectomy or transurethral resection of the prostate (TURP) depending on patient׳s diagnosis).
Experimental features	Transcriptomic libraries were constructed using Ion Total RNA-Seq Kit v2 (Life Technologies). Excessive rRNA was removed using duplex-specific nuclease (DSN) normalization method. Targeted DNA enrichment was performed by GeneRead DNAseq Targeted Human Prostate Cancer Panel.
Data source location	Moscow, Russia
Data accessibility	Raw data was deposited at NCBI SRA database under accession No. SRP093707https://www.ncbi.nlm.nih.gov/bioproject/PRJNA354519 and SRP093842https://www.ncbi.nlm.nih.gov/bioproject/PRJNA354799.

**Value of the data**

• The data on protein coding and non-coding RNAs detectable in urine and plasma of patients with PCa is valuable for the screening of novel biomarkers for early diagnostics of PCa.
• Targeted DNA sequencing data can be used to develop methods of revealing resistance to commonly used anti-androgen therapy at the early stages of PCa progression.
• Both datasets include samples obtained from patients with BPH which can be used as a control group to ensure high specificity of found markers towards PCa (Nikitina et al., 2016) [1].
• Access to the raw sequencing data allows researchers to perform further bioinformatic analysis based on their own computational algorithms.

## 1 Experimental design, materials and methods

### 1.1 Sample collection, RNA and DNA extraction

Urine and plasma samples were taken from 14 patients with PCa and 3 patients with BPH from Moscow City Clinical Hospital No. 50. Urine samples were obtained after prostate massage. Each urine and plasma sample was collected either before or after the operation (radical prostatectomy or transurethral resection of the prostate (TURP) depending on patient׳s diagnosis). RNA and DNA extractions from urine were performed using RNA/DNA Purification Kit (Norgen Biotek). Isolation of these molecules from plasma was carried out by QIAamp Circulating Nucleic Acid Kit (Qiagen). Overall, 19 RNA (12 from urine and 7 from plasma) samples and 41 DNA (14 from urine and 27 from plasma) samples were obtained. Table 1 provides information about patients and the samples obtained from them. Each sample name corresponds to a single library and to a single FASTQ record in NCBI SRA database.

### 1.2 Transcriptome library preparation

DNA contaminations from extracted total RNA were removed with the use of DNAse I (Fermentas) treatment following the manufacturer׳s recommendations. RNA concentration was determined by fluorometer Qubit 2.0 using Qubit RNA HS Assay Kit (Thermo Fisher). Transcriptomic libraries were constructed using Ion Total RNA-Seq Kit v2 (Life Technologies) with the following modifications of the protocol. For RNA fragmentation 1 µl of 10x RNase III buffer (Life Technologies) was added to 9 µl of RNA solution and heated for 10 min at 95 °С followed by immediate snap-cooling on ice. After that 1 µl of 10 µM ATP and 1 µl of polynucleotide kinase (Fermentas) were added to the solution from the previous step and the whole mix was incubated at 37 °С for 30 min. Fragmented RNA was cleaned up using Micro Bio-Spin Chromatography Columns (Bio-Rad). Further steps of library preparation including adapter ligation, first-strand cDNA synthesis, and amplification were carried out in accordance with manufacturer׳s instructions. Fragments of cDNA corresponding to rRNA were depleted using duplex-specific nuclease (Evrogen) as described in [2] with the following modifications. Only one round of DSN-normalisation was performed and the re-annealing of ds-cDNA at 68 °C was carried out overnight. The prepared library was purified by magnetic beads Agencourt AMPure XP (Beckman Coulter Inc.) and its quality was assessed by 2100 Bioanalyzer (Agilent Genomics) using Agilent High Sensitivity DNA Kit (Agilent Genomics).

### 1.3 Targeted DNA library preparation

GeneRead DNAseq Targeted Human Prostate Cancer Panel (Qiagen) was used for targeted enrichment of the extracted DNA. Considering the quality of circulating cell-free DNA the number of cycles in this amplification step was raised to 20 and 22 for DNA extracted from urine and plasma respectively. Subsequent library construction was performed using GeneRead Library Prep workflow (Qiagen) following the manufacturer׳s recommendations.

### 1.4 High-throughput sequencing

The sequencing of constructed libraries was performed on Ion Proton platform using ION PI HI-Q Sequencing 200 Kit and Ion PI Chip Kit v2 (Thermo Fisher Scientific) following the recommendations of the manufacturer. Base calling was performed by Torrent Suite 5.0, fastqCreator v3.4.56313.

## Figures and Tables

**Table 1:** Sample information.

**Patient info**			**Sample names**							
**Patient ID**	**Diagnosis**	**Age**	**RNA**				**DNA**			
			**Urine**		**Plasma**		**Urine**		**Plasma**	
			**Before**	**After**	**Before**	**After**	**Before**	**After**	**Before**	**After**
A50_001	BPH	75	PC112R			PC307R			PC312D	PC307D
A50_002	BPH	67		PC107R			PC111D	PC107D	PC311D	PC306D
A50_003	BPH	58	PC113R				PC113D		PC313D	
A50_006	PCa	64	PC116R							
P50_001	PCa	60		PC148R		PC348R			PC321D	PC348D
P50_002	PCa	55							PC322D	PC349D
P50_003	PCa	61					PC123D	PC150D	PC323D	PC350D
P50_004	PCa	55	PC124R				PC124D		PC324D	PC351D
P50_006	PCa	67	PC126R		PC326R	PC353R	PC126D		PC326D	PC353D
P50_008	PCa	69					PC128D	PC102D	PC328D	PC302D
P50_009	PCa	57					PC108D		PC308D	PC303D
P50_010	PCa	69	PC109R	PC104R	PC309R	PC304R	PC109D		PC309D	PC304D
P50_013	PCa	56					PC130D		PC330D	PC355D
P50_015	PCa	48	PC132R				PC132D		PC332D	PC357D
P50_020	PCa	50					PC137D		PC337D	PC362D
P50_032	PCa	56	PC183R							
P50_033	PCa	60	PC185R							
