# Supplementary material for: Datasets for next-generation sequencing of DNA and RNA from urine and plasma of patients with prostate cancer
Source: Data Brief. 2016 Dec 14;10:369–72. doi: 10.1016/j.dib.2016.12.016 (PMC5175992; doi:10.1016/j.dib.2016.12.016)
Supplement: Supplementary file 1 — Supplementary material [file mmc1.docx]

The authors declare no conflict of interests.
